# Preliminary Clinical Experience of *trans*-1-Amino-3-(18)F-fluorocyclobutanecarboxylic Acid (*anti*-(18)F-FACBC) PET/CT Imaging in Prostate Cancer Patients

**DOI:** 10.1155/2014/305182

**Published:** 2014-06-01

**Authors:** Kalevi Kairemo, Nigora Rasulova, Kaarina Partanen, Timo Joensuu

**Affiliations:** ^1^Department of Molecular Radiotherapy & Nuclear Medicine, Docrates Cancer Center, Saukonpaadenranta 2, 00180 Helsinki, Finland; ^2^Department of Radiology, Docrates Cancer Center, Saukonpaadenranta 2, 00180 Helsinki, Finland; ^3^Department of Medical Oncology and Clinical Radiotherapy, Docrates Cancer Center, Saukonpaadenranta 2, 00180 Helsinki, Finland

## Abstract

*Background.* In this retrospective analysis we assessed the role of [^18^F]-FACBC-PET/CT in the prostatic cancer staging. *Procedure.* 30 first [^18^F]-FACBC-PET/CT images of 26 patients (68.1 ± 5.8 years) were analyzed. PET/CT findings were compared with PSA concentrations, with PSA doubling times (PDT), and with correlative imaging. *Results.* On 16 [^18^F]-FACBC (53.3%) scans, 58 metabolically active lesions were found. 12 (20.7%) lesions corresponding to the local relapse were found in prostate/prostate bed and seminal vesicles, 9 (15.5%) lesions were located in regional lymph nodes, 10 (17.2%) were located in distal lymph nodes, and 26 (44.8%) metabolically active lesions were found in the skeleton. In one case, focal uptake was found in the brain, confirmed further on MRI as meningioma. The mean S-PSA level in patients with positive [^18^F]-FACBC findings was 9.5 ± 16.9 **μ**g/L (0.54–69 **μ**g/L) and in patients with negative [^18^F]-FACBC findings was 1.96 ± 1.87 **μ**g/L (0.11–5.9 **μ**g/L), but the difference was not statistically significant. However, the PSA doubling time (PDT) in patients with positive findings was significantly shorter than PDT in patients with negative findings: 3.25 ± 2.09 months (0.3–6 months) versus 31.2 ± 22.02 months (8–84 months), *P* < 0.0001. There was a strong positive correlation between PSA value and number of metabolically active lesions (*R* = 0.74) and a negative correlation between PDT and number of metabolically active lesions (*R* = −0.56). There was a weak negative correlation between PDT and SUV_max⁡_ (*R* = −0.30). *Conclusion.* According to our preliminary clinical experience, [^18^F]-FACBC-PET may play a role in *in vivo* restaging of an active prostate cancer, especially in patients with a short S-PSA doubling time.

## 1. Introduction

Trans-1-amino-3-^18^F-fluorocyclobutanecarboxylic  acid (anti-[^18^F]-FACBC) is an amino acid positron emission tomography (PET) tracer that has shown promise for visualizing prostate cancer. This [^18^F]-FACBC tracer was developed for L-amino acid transport evaluation; it demonstrated favorable dosimetry, liver being the critical organ, and 14 *μ*Sv/MBq effective dose was measured in 6 healthy volunteers in a first-in-man study [1]. Its safety, tracer stability, and uptake kinetics in patients have been reported in a phase I trial [2].

This tracer has been used in 10 prostate cancer patients before prostatectomy to find lesions in different prostate regions [3] and in another 10 patients to successfully separate malignant lung lesions from inflammatory pulmonary lesions [4].

In the literature, clinical results about its targeting ability in prostate cancer have also been superior as compared to Prostascint [5] and as compared to [^11^C]-choline [6, 7]. Anti-3-[^18^F]-FACBC demonstrated a sensitivity of 89% (36 patients) for cancer in the prostate bed and a sensitivity of 100% for detecting an extraprostatic recurrence (10 patients) [5].

[^18^F]-FACBC-PET is believed to be useful not only for the visualization of human prostate cancer, but also for differentiating cancer from inflammation and from benign hyperplasia as studied in animals. Also, most of the mechanisms are known from cellular studies [8].

FACBC is not yet a registered radiopharmaceutical either in EU or in the US. Fluorocholine (FCH) is a registered radiopharmaceutical in a few European countries, such as France, and it is considered currently the standard imaging agent for staging prostate cancer. According to the largest meta-analysis so far [9], the specificity of [^18^F]-FCH-PET is high, but the sensitivity is low. Very little is known yet about the intraprostatic distribution of FCH and its role in T-staging [9].

The mechanism of [^18^F]-FACBC uptake is different as compared to that of fluorocholine. Both tracers are anabolic, but [^18^F]-FACBC is active in amino acid transport for protein synthesis, whereas FCH is participating in the phosphatidylcholine synthesis necessary for cell membrane renewal.

In cell lines, it has been shown that [^18^F]-FACBC is not incorporated into proteins in prostate cancer cells; and (Na+)-dependent L-amino acid transport system is mainly responsible for the active mechanism of cellular uptake, whereas (Na+)-independent transport mechanisms do not participate in cellular uptake [10]. In rat orthotopic model, this transport mechanism has been confirmed using the same cell line DU 145 as in the cellular mechanism studies [10]. Additionally, in a dual disease animal model both for inflammation and prostate cancer, it could be shown that [^18^F]-FACBC targets human prostate cancer and could separate cancer from inflammation [8]. However, all clinical reports are preliminary so far, and all investigators warrant further studies in clinical trials. The present analysis is the full report of our experience in 30 first clinical imaging examinations. Our main purpose in the present paper was to analyze the clinical suitability of [^18^F]-FACBC PET/CT for restaging of biochemical relapse in radically treated prostate cancer.

## 2. Materials and Methods

### 2.1. Patients

30 [^18^F]-FACBC-PET/CT scans of 26 patients (25 with confirmed prostatic cancer in the period 1999–2013) were analyzed: age ranging from 56 to 77 years (mean age 68.1 ± 5.8); Gleason score 5–9 (mean 7.1 ± 1.4); PSA value 0.11–69 *μ*g/L (mean 7.9 ± 14.6). FACBC-PET/CT scans for restaging were done in 25 patients with confirmed prostatic cancer and in one patient with suspicion of prostate cancer.

12 patients had radical prostatectomy and following radiotherapy with a dose of 66–77 Gy, 13 patients received radical radiotherapy 66–77 Gy. 20 patients were receiving androgen deprivation therapy (ADT), 11 bisphosphonate therapy, 5 chemotherapy, and 7 Sm-153-EDTMP therapy, and 1 additionally had received denosumab (Xgeva). And only one patient was suspected for prostate cancer due to increased PSA level. The initial staging and the received treatments by patients are listed in Table 1.

This work is a retrospective analysis of our thirty first [^18^F]-FACBC-PET/CT images. [^18^F]-FACBC was used with a special permission for compassionate use from the Finnish Medical Evaluation Agency (FIMEA permission number 14863/2012) for individual cancer management purposes at our institution. This analysis was performed according to the principles of the Declaration of Helsinki. Written informed consent was obtained from all patients, and our patient database was approved by the Finnish authority for the protection of privacy and personal data in clinical research.

### 2.2. Imaging PET/CT Protocol

Examination was done on Siemens Biograph PET Scanner, combined with low-dose CT. The injected activity dose of [^18^F]-anti-FACBC ranged from 230 to 320 MBq (mean 328 ± 56.8). Early imaging was performed by starting at 8.0 ± 3.0 min (range of 4–16 min) from pelvic region using 3 min per bed position imaging protocol followed by whole body imaging from the calvarium to the midthighs at 23.4 ± 6.3 min (range of 16–37 min) using 3 min per bed position. The tracer was purchased from Uppsala (Uppsala Imanet, Sweden), and the production has been described by Sörensen et al. [2].

### 2.3. Image Analysis

Lesions were considered abnormal when focal tracer accumulation was more than 30% greater than normal organ activity. (1) Diagnosis of malignant lymph nodes on [^18^F]-FACBC-PET images was based on visual assessment. (2) Lymph nodes were considered benign if they were not larger than 10 mm on CT scans and without abnormal [^18^F]-FACBC uptake. (3) Interpretation of malignant or benign bone lesions depended on the anatomical localization, and the presence/absence of [^18^F]-FACBC uptake was compared with the findings of correlative imaging, such as CT, MRI, Na [^18^F]-F-PET/CT, or [^18^F]-FCH-PET/CT. The correlative imaging was counted if performed within 2 weeks from the [^18^F]-FACBC-PET/CT. These images were based on our clinical practice, not in any protocols. (4) In the prostate tissue, SUV_max⁡_-value 3.5 (mg/mL) was considered as cut-off limit. Additionally, an increasing uptake to the late image from the early image in the prostate region was classified as pathological.

### 2.4. Statistical Analysis

The acquired results were expressed as the mean ± SEM for each variable. Comparison of data among various groups was performed with Student's unpaired* t*-test. A *P* < 0.05 was considered statistically significant. For calculating correlation between PSA, PSA doubling time DT (PDT), SUVs, and number of metabolically active lesions, Spearman rank correlation coefficient and simple linear regression for building the curves were used. In case of non-Gaussian distribution Mann-Whitney* U* test was used.

## 3. Results

On 16 [^18^F]-FACBC-PET scans (53.3%), a total of 58 metabolic active lesions were detected. In prostate, prostate bed and seminal vesicles focal uptake was found in 12 cases (20.7%), with SUV_max⁡_ ranging from 3.7 to 7.2 (mean 5.07 ± 1.9). In local lymph nodes, 9 lesions (15.5%) were found with SUV_max⁡_ 4.1–10.7 (mean 8.1 ± 2.6) and, in distal lymph nodes, 10 lesions (17.2%) with SUV_max⁡_ 3.4–11.9 (mean 7.35 ± 2.7) were found. A total of 26 bone lesions (44.8%) with SUV_max⁡_ 4.2–8.8 (mean 5.4 ± 0.7) were observed. In one case, there was increased tracer uptake in the brain with a SUV_max⁡_  8.1 (1.7%) confirmed later on MRI as meningioma. The findings are presented for each patient individually in Table 1.

The mean PSA level in patients with negative [^18^F]-FACBC-PET findings was 1.96 ± 1.87 *μ*g/L (0.11–5.99), whereas, with [^18^F]-FACBC positive PET findings, it was 9.5 ± 16.9 *μ*g/L (0.54–69). There was no statistically significant difference in the PSA values between patients with positive and negative findings (*P* < 0.2, Mann-Whitney* U* test). However, there was statistically significant difference in the PDTs in patients with positive findings 3.25 ± 2.09 months (0.3–6 months) versus PDTs in patients with negative findings 31.2 ± 22.02 months (8–84 months) (*P* < 0.0001) (Table 2).

**Figure 1:**
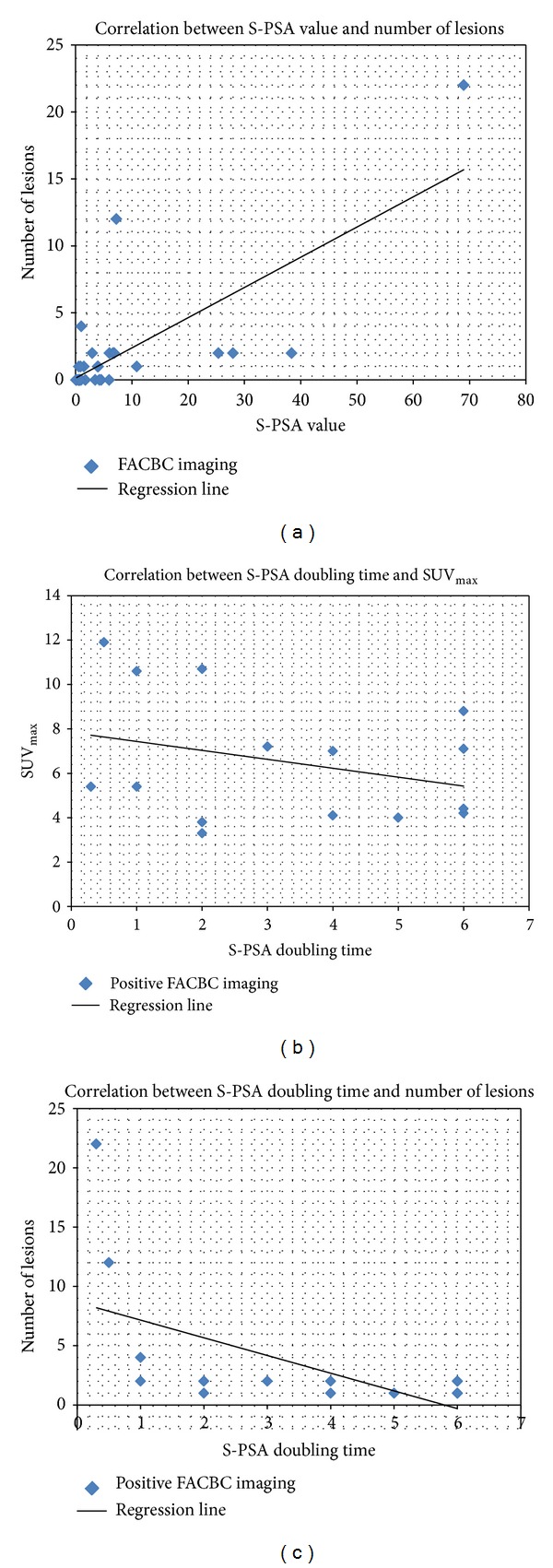
Correlations between the PSA value and the number of lesions (a), between S-PSA doubling time and SUV max of the FACBC study (b), and between the number of metabolically active lesions and the PSA doubling time (c) are shown schematically. There is a strong correlation between number of metabolically active lesions and PSA (*R* = 0.74) and weaker negative correlations between number of lesions and PSA-doubling time (*R* = −0.56) and between SUV_max⁡_ and PSA-doubling time (*R* = −0.30).

The correlations between PSA value and number of lesions, between PDT and number of lesions and between PDT and SUV_max⁡_, are shown in Figure 1.

**Figure 2:**
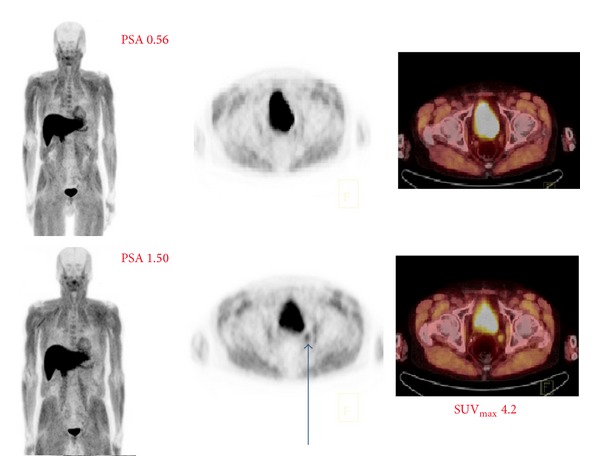
A 70-year-old patient had Gleason score 9 disease (T3bN0M0) treated with radical prostatectomy 6 years earlier and he developed a biochemical relapse. The first investigation was negative at serum PSA concentration 0.56, but, in the second examination 3 months later at PSA concentration 1.50, a small lymph node uptake was found in an obturator lymph node (SUV_max⁡_ 4.2); retrospectively, there was no significant uptake (SUV_max⁡_ 1.7) in the first scanning. Normal distribution is seen in the liver, pancreas, skeletal muscles, and also in the urinary bladder.

**Figure 3:**
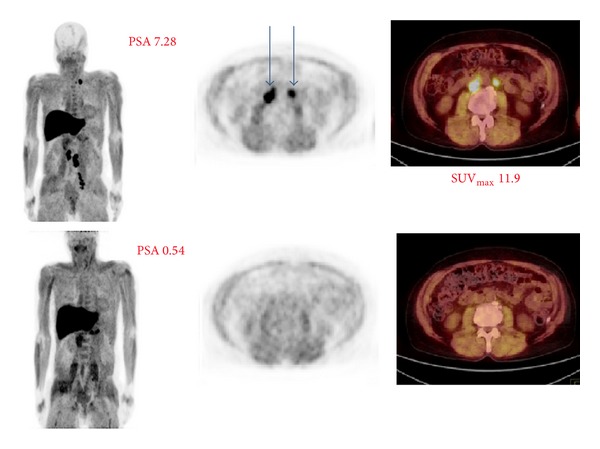
67-year-old patient had Gleason score 9 disease (T4N1M1) treated originally with a radiation therapy 4 years earlier. After that, he had antiandrogen treatment but developed a biochemical relapse. The first examination was positive at serum PSA concentration 7.28, but, in the second scanning 3 months later, became negative when the PSA concentration was 0.54. The first imaging demonstrated a large tumor burden in left iliac, para-aortic, aortocaval, and left supraclavicular lymph nodes; SUV_max⁡_ levels were up to 11.9. All these lymph node uptakes disappeared in 3 months as a treatment response to chemotherapy, and no pathologic findings were seen in the second imaging.

To illustrate the [^18^F]-FACBC-PET in clinical practice, two patients with multiple diagnostic imaging are presented. The patient (number 10 in Table 1) had a totally negative study at serum PSA concentration 0.56, which turned positive 3 months later at serum PSA concentration 1.50 (Figure 2). Another patient (number 13 in Table 1) shows a successful treatment response evaluation by [^18^F]-FACBC-PET/CT follow-up (Figure 3).

## 4. Discussion

This synthetic FACBC amino acid, which is an isoleucine analogue, is developed for assessment of the anabolic component of tumor metabolism in clinical routine PET. The uptake of FACBC is mediated by the large-neutral amino acid transport system, and it is transported into cells but is not incorporated into proteins [8]. Because only a small amount of FACBC is excreted into the urinary system and because amino acid uptake is enhanced in malignancies, FACBC may play an essential role in the detection of prostate cancer. Its clinical utility is still unknown [11].

In the retrospective analysis of our clinical data, [^18^F]-FACBC-PET showed its capability of targeting active prostate cancer in patients. Our patient numbers are still low, but our results support the previous findings [2–7]. The specificity seems to be high in these studies.

Results about characterizing prostate tissue* in vivo* have been reported in 10 patients with prostate carcinoma studied using [^18^F]-FACBC before prostatectomy [3]. In these patients, surgical specimen analysis was compared with the dynamic PET imaging results. 79 sextants had malignancy and 41 were benign: SUV_max⁡_ was significantly higher (*P* < 0.05) in malignant sextants (e.g., 4.0 ± 1.3 at 28 min) compared to nonmalignant sextants (e.g., 3.4 ± 0.9 at 28 min), although there was overlap between malignant and nonmalignant sextants. SUV_max⁡_ also significantly correlated (*P* < 0.05) with Gleason score at all imaging time points (e.g., *r* = 0.46 at 28 min). Since there was no distinct separation between malignant and nonmalignant sextants or between Gleason score levels, the authors thought that FACBC alone was not good enough for radiation therapy planning but may be useful to guide the biopsy of the most aggressive lesion [3]. Similarly, surgical specimen analysis was performed in lung lesions in 10 patients [4], and anti-3-[^18^F]FACBC uptake in malignant lesions (SUV_max⁡_5.9 ± 3.4) was greater than that in inflammatory lesions (SUV_max⁡_2.2 ± 0.03) at 28 min (*P* < 0.05).

In our analysis, the S-PSA level correlated strongly with the number of metabolically active lesions to be detected on PET. Additionally, there was a moderate negative correlation between PDT and number of lesions and a weak negative correlation between PDT and tumor activity on PET (SUV_max⁡_). There was not any statistically significant difference between S-PSA values in groups of patients with negative and positive scans.

However, the PDT in patients with positive findings comparing to negative ones was statistically significantly shorter 3.25 ± 2.09 months versus 31.2 ± 22.02 months, *P* ≤ 0.0001. In the work of Ceci et al. [12], in patients with relapse, detected by [^11^C]-choline PET/CT, the median PSA doubling time was 3.5 months and the mean PSA level was 9.08 *μ*g/L ± 5.1 *μ*g/L, with a range of 2–60 ng/mL. In our analysis, the mean PSA level was 12.8 ± 18 *μ*g/L with a range of 0.62–69 *μ*g/L. From these results, it seems that the PSA doubling time is a more important prognostic factor than PSA level.

We know already from this preliminary experience that PSA levels can be low for positive scans. The patient in Figure 2 was definitely positive when PSA was 1.50 but doubtful already at the level 0.56 indicating the rapid repeated diagnostic procedure. In the literature, a [^18^F]-FACBC uptake in a pelvic lymph node on PET as a sign of biochemical relapse has been reported with a PSA level as low as 0.03 *μ*g/L in a prostatectomized patient [13].

Anti-3-[^18^F]-FACBC demonstrated for disease detection in the prostate bed a sensitivity of 89% (32 of 36 patients), specificity of 67% (8 of 12 patients), and accuracy of 83% (40 of 48 patients) [5]. In the detection of an extraprostatic recurrence, anti-3-[^18^F]-FACBC had a sensitivity of 100% (10 of 10 patients) [5].

There is only one comparison in the literature, between [^18^F]-FACBC and [^11^C]-choline, but no clear conclusions can be drawn [6, 7]. The existing trends in the study favor FACBC in diagnosing prostate cancer. Anyhow, the radionuclides C-11 and F-18 have essential differences, for example, in chemical and physical characteristics. In order to understand the biochemical behavior better, FCH should be compared with FACBC in clinical trials, ideally. In the existing comparisons [6, 7], the patient preparation such as fasting time was a little bit different for different tracers; very few biopsies and surgeries were performed only in 5 patients and no statistical difference was found. Many patients had also androgen deprivation therapy on hold, but some had not [6, 7]. [^11^C]-choline has shown its capability in restaging studies in predicting prostate cancer survival in a large multicenter study in prostatectomized patients during androgen deprivation therapy [14].

In our material, very few patients were imaged with both [^18^F]-FACBC and [^18^F]-FCH within the 2-week interval, but many patients had previous and follow-up [^18^F]-FCH-PET/CT studies with longer intervals. At least in two patients, we observed that some pharmaceuticals may influence the FCH uptake, but we have not yet seen any effect on FACBC uptake. Some of these effects with FCH have been reported [15]. We had two patients with false positive [^18^F]-FACBC findings in the prostate. Both patients (patients 23 and 26, Table 1) had negative findings in all 12 prostate biopsies interpreted as inflammatory and reactive changes to EBRT and all immunohistochemical staining for prostate cancer were negative. Patient 23 was negative in [^18^F]-FCH-PET study two months earlier.

From our experience, the regular distribution of [^18^F]-FACBC in man demonstrating an intense uptake in the pancreas and in the liver and a lesser activity in the bone marrow is actually almost ideal for prostate cancer detection, because these regions are usually free from the disease in early stage. Sometimes, skeletal muscle uptake may disturb differential diagnosis, but delayed imaging may help. The optimal time for highest tumor/nontumor ratio is at 28 min as reported by [3]. Urinary clearance appears seldom, even though a little urinary excretion is seen like in our patient in Figure 2.

From this preliminary data, we conclude that [^18^F]-FACBC-PET may play a role in in vivo staging of an active prostate cancer. Although, according to our preliminary experience, there was no statistically significant difference in PSA level between the patients with positive and negative findings; short PSA doubling time may be indicator for a PET/CT study. We are aiming to use [^18^F]-FACBC-PET in dose planning for external beam radiation therapy and in normal diagnostic staging procedures, especially in restaging of biochemical relapse.

## Figures and Tables

**Table 1:** Patient's characteristics and summary of FACBC-PET/CT, correlative imaging, and histopathology results. ( ) refer to number of lesions, (+) to positive findings, and (−) to negative findings.

No.	Age	Diagnosed	Gleason/Grade	TNM	Surgery	Radiotherapy	ADT	Bisphosphonates	Chemotherapy	Sm-153-EDTMP	FACBC	Correlative findings	Comments
1	67	Suspicion	No cancer	—	—	—	—	—	—	—	CNS (1) no other findings	MRI prostate biopsy (−)	Meningioma FP S-PSA 4.05 TN
2	71	2006	6/1	T1aN0	—	X	—	—	—	—	No findings	NaF (−), FCH (−)	S-PSA 4.58 TN
3	75	2008	7/2	T3N0	—	X	X	X	X	—	Skeletal (1)	NaF (+)	S-PSA 3.97 TP
4	73	1999	5/2	T3N0	—	X	—	—	—	—	No findings	NaF (−), F/U (−)	S-PSA 0.70 TN
5	72	1999	5/2	pT1cN0	X	X	X	—	—	—	No findings	—	S-PSA 5.99
6	60	2010	7/2	pT2bN0	X	X	X	—	—	—	Lnn L (1), lnn D (1)	CT (+)	S-PSA 25.4 TP
7	64	2005	9/3	pT2aN0	X	X	X	—	—	—	No findings	F/U (−)	S-PSA 1.75 TN
8	73	1999	6/2	T3-4Nx	—	X	X	X	—	—	Lnn D (2)	FCH (+), CT (+)	S-PSA 38.4 TP
9	66	2013	7/3	T1cN0	—	X	X	—	—	—	Prostate (1), lnn L (1)	prostate biopsy (+), F/U (+)	S-PSA 28.0 TP
10.1	70	2007	9/3	pT2Nx	X	X	X	X	—	X	No findings	CT (−)	S-PSA 0.56 TN
10.2	70	2007	9/3	pT2Nx	X	X	X	X	—	X	Lnn L (1)	F/U (+)	S-PSA 1.50 TP
11	65	2003	6/2	pT1cN0	X	X	—	—	—	—	No findings	F/U (−)	S-PSA 0.76 TN
12	69	2004	5/1	T1cN0	—	X	X	—	—	—	No findings	F/U (−)	S-PSA 3.51 TN
13.1	67	2009	9/3	T4N1	—	X	X	X	X	X	Prostate (1), lnn L (1), lnn D (1), skeletal (1)	NaF (+), CT (+)	S-PSA 1.04 TP
13.2	67	2009	9/3	T4N1	—	X	X	X	X	X	Prostate (1), vesicle (2), lnn L (4), lnn D (5), skeletal (0)	F/U (+/−), response to local RT to bone lesion	S-PSA 7.28 TP/TN
13.3	67	2009	9/3	T4N1	—	X	X	X	X	X	Prostate (0), vesicle (0), lnn L (0), lnn D (0), skeletal (0)	F/U (−) complete response to chemo- and RT	S-PSA 0.54 TN
14	67	2004	6/2	pT2bN0	X	X	X	X	—	X	No findings	F/U (−)	S-PSA 0.62 TN
15.1	76	2000	7/2	pT3bN0	X	X	X	—	—	—	No findings	F/U (−)	S-PSA 0.23 TN
15.2	76	2000	7/2	pT3bN0	X	X	X	—	—	—	Prostate bed (1)	F/U (+)	S-PSA 0.62 TP
16	56	2013	6/2	T3bN1	—	X	—	—	—	—	Prostate (2)	Prostate biopsy (−/+), MRI (+/−), F/U (+)	S-PSA 6.68 FP/TP
17	76	2001	7/2	pT3N0	X	X	X	—	—	—	Skeletal (22)	FCH (+), F/U (+)	S-PSA 69.0 TP
18	58	2012	7/2	pT3Nx	X	X	X	—	—	—	No findings	—	S-PSA 1.00
19	72	2007	7/2	T3bN1	—	X	X	X	X	X	Skeletal (2)	FCH (+), F/U (+), MRI	S-PSA 3.00 TP
20	59	2009	8/3	pT3Nx	X	X	X	X	X	—	No findings	F/U (−)	S-PSA 0.11 TN
21	69	2000	6/2	pT1cNx	X	X	X		X	—	Lnn L (1), lnn D (1)	FCH (+), MRI (+)	S-PSA 6.06 TP
22	69	2009	8/3	T3N3	—	X	X	X	—	X	No findings	—	S-PSA 0.78
23	77	2012	9/3	T3bN1	—	X	X	X	—	X	Prostate (1)	Prostate biopsy (−) F/U (−)	S-PSA 10.9 FP reactive changes
24	61	2007	6/1	T1cN0	—	X	X	X	—	X	Prostate (2)	F/U (+), FCH (+)	S-PSA 6.90 TP
25	74	2010	9/3	T3bN0	—	X	X	X	—	—	Prostate (1)	F/U (+)	S-PSA 0.91 TP
26	66	2009	8/3	T2N3	—	X	—	—	—	—	No findings	Prostate biopsy (−) F/U (−)	S-PSA 4.26 TN prostatitis

Abbreviations: F/U-follow up, CT: computer tomography, NaF: sodium fluoride PET/CT, FCH: fluorocholine PET/CT, lnn: lymph nodes, L: local, D: distal, TP: true positive, TN: true negative, and FP: false positive.

Surgery-radical prostatectomy ± pelvic lymphadenectomy, radiotherapy 68–78 Gy on prostate ± lymph nodes. ADT: androgen deprivation therapy.

Patient 14 had Denosumab (XGEVA).

**Table 2:** PSA level and PSA doubling time in group of patients with positive and negative FACBC findings.

Studies with positive [^18^F]-FACBC findings (I) (*n* = 15*)		Studies with negative [^18^F]-FACBC findings (II) (*n* = 15*)	
S-PSA level	S-PSA doubling time**	S-PSA level	S-PSA doubling time**

9.5 ± 16.9 (0.54–69 *μ*g/L)	3.25 ± 2.09 months (0.3–6 months)	1.96 ± 1.87 *μ*g/L (0.11–5.99 *μ*g/L)	31.2 ± 22.02 months (8–84 months)

*Patient 1 with no cancer was considered negative.

**Statistically significant, *P* ≤ 0.0001.

## References

[1] Nye JA, Schuster DM, Yu W, Camp VM, Goodman MM, Votaw JR (2007). Biodistribution and radiation dosimetry of the synthetic nonmetabolized amino acid analogue anti- 18F-FACBC in humans. *Journal of Nuclear Medicine*.

[2] Sörensen J, Owenius R, Lax M, Johansson S (2013). Regional distribution and kinetics of [18F]fluciclovine (anti-[18F]FACBC), a tracer of amino acid transport, in subjects with primary prostate cancer. *European Journal of Nuclear Medicine and Molecular Imaging*.

[3] Schuster DM, Taleghani PA, Nieh PT (2013). Characterization of primary prostate carcinoma by anti-1-amino-2-[(18)F] -fluorocyclobutane-1-carboxylic acid (anti-3-[(18)F] FACBC) uptake. *The American Journal of Nuclear Medicine and Molecular*.

[4] Amzat R, Taleghani P, Miller DL (2013). Pilot study of the utility of the synthetic PET amino-acid radiotracer anti-1-amino-3-[18F]fluorocyclobutane-1-carboxylic acid for the noninvasive imaging of pulmonary lesions. *Molecular Imaging and Biology*.

[5] Schuster DM, Savir-Baruch B, Nieh PT (2011). Detection of recurrent prostate carcinoma with anti-1-amino-3- 18F-fluorocyclobutane-1-carboxylic acid PET/CT and 111In-capromab pendetide SPECT/CT. *Radiology*.

[6] Nanni C, Schiavina R, Boschi S (2013). Comparison of 18F-FACBC and 11C-choline PET/CT in patients with radically treated prostate cancer and biochemical relapse: preliminary results. *European Journal of Nuclear Medicine and Molecular Imaging*.

[7] Nanni C, Schiavina R, Brunocilla E (2014). 18F-FACBC compared with 11C-Choline PET/CT in patients with biochemical relapse after radical prostatectomy: a prospective study in 28 patients. *Clinical Genitourinary Cancer*.

[8] Oka S, Hattori R, Kurosaki F (2007). A preliminary study of anti-1-amino-3-18f-fluorocyclobutyl-1- carboxylic acid for the detection of prostate cancer. *Journal of Nuclear Medicine*.

[9] von Eyben FE, Kairemo K (2014). Meta-analysis of 11C-choline and 18F-choline PET/CT for management of patients with prostate cancer. *Nuclear Medicine Communications*.

[10] Okudaira H, Shikano N, Nishii R (2011). Putative transport mechanism and intracellular fate of trans-1-amino-3-18F-fluorocyclobutanecarboxylic acid in human prostate cancer. *Journal of Nuclear Medicine*.

[11] Castellucci P, Jadvar H (2012). PET/CT in prostate cancer: Non-choline radiopharmaceuticals. *Quarterly Journal of Nuclear Medicine and Molecular Imaging*.

[12] Ceci F, Castellucci P, Graziani T (2014). 11C-Choline PET/CT detects the site of relapse in the majority of prostate cancer patients showing biochemical recurrence after EBRT. *European Journal of Nuclear Medicine and Molecular Imaging*.

[13] von Eyben FE, Kangasmäki A, Kiljunen T, Joensuu T (2013). Volumetric-modulated arc therapy for a pelvic lymph node metastasis from prostate cancer: a case report. *Tumori*.

[14] Giovacchini G, Picchio M, Garcia-Parra R (2014). 11C-Choline PET/CT predicts prostate cancer-specific survival in patients with biochemical failure during androgen-deprivation therapy. *Journal of Nuclear Medicine*.

[15] Beheshti M, Haim S, Zakavi R (2013). Impact of 18F-choline PET/CT in prostate cancer patients with biochemical recurrence: influence of androgen deprivation therapy and correlation with PSA kinetics. *Journal of Nuclear Medicine*.

